# Biomechanical Comparison between Isobar and Dynamic-Transitional Optima (DTO) Hybrid Lumbar Fixators: A Lumbosacral Finite Element and Intersegmental Motion Analysis

**DOI:** 10.1155/2022/8273853

**Published:** 2022-07-08

**Authors:** Shih-Hao Chen, Chih-Kun Hsiao, Chih-Wei Wang, Hsiang-Ho Chen, Zheng-Cheng Zhong

**Affiliations:** ^1^Department of Orthopaedics, Tzu Chi General Hospital at Dalin and Tzu Chi University, Taiwan; ^2^Department of Orthopedics, E-Da Hospital, Kaohsiung, Taiwan; ^3^Center for Biomedical Engineering, Department of Biomedical Engineering, College of Engineering, Chang Gung University, Taoyuan 33302, Taiwan; ^4^Department of Mechanical Engineering, National Yang Ming Chiao Tung University, Hsinchu, Taiwan

## Abstract

Biomechanical performance of longitudinal component in dynamic hybrid devices was evaluated to display the load-transfer effects of Dynesys cord spacer or Isobar damper-joint dynamic stabilizer on junctional problem based on various disc degenerations. The dynamic component was adapted at the mildly degenerative L3–L4 segment, and the static component was fixed at the moderately degenerative L4–L5 segment under a displacement-controlled mode for the finite element study. Furthermore, an intersegmental motion behavior was analyzed experimentally on the synthetic model under a load-controlled mode. Isobar or DTO hybrid fixator could reduce stress/motion at transition segment, but compensation was affected at the cephalic adjacent segment more than the caudal one. Within the trade-off region (as a motion-preserving balance between the transition and adjacent segments), the stiffness-related problem was reduced mostly in flexion by a flexible Dynesys cord. In contrast, Isobar damper afforded the effect of maximal allowable displacement (more than peak axial stiffness) to reduce stress within the pedicle and at facet joint. Pedicle-screw travel at transition level was related to the extent of disc degeneration in Isobar damper-joint (more than Dynesys cord spacer) attributing to the design effect of axial displacement and angular rotation under motion. In biomechanical characteristics relevant to clinical use, longitudinal cord/damper of dynamic hybrid lumbar fixators should be designed with less interface stress occurring at the screw-vertebral junction and facet joint to decrease pedicle screw loosening/breakage under various disc degenerations.

## 1. Introduction

Spinal fusion with static fixation has been used for the treatment of lumbosacral instability caused by degenerative disorders. The high rigidity-raising effect of pedicle static fixators inevitably induces a potential culprit in developing junctional problems attributed to the overconstrained mobility of fixed segments and more load being compensated by the adjacent segments [1–3]. Pedicle-based dynamic stabilization (PDS) adapts the potentials of internal bracing pathologic spinal segments to restore near-normal kinematic behavior, thus mitigating the adjacent level effect and iatrogenic degeneration [4–6]. The Dynesys dynamic stabilization system (Zimmer Inc., Warsaw, IN, USA) contains unique spacers made of cannulated polycarbonate urethane and tensioned cords made of polyethylene terephthalate. It forms a screw spacer and spacer cord linkage to provide motion preservation and load-protecting abilities at the bridged segments [7–10]. Recently, the hybrid use of “dynamic-transitional optima” (DTO) has been applied with the unstable segments being fixed by a static fixator and the transition segments further bridged by a dynamic Dynesys fixator [10–13]. On the other hand, the Isobar TTL system (Scient'x USA, Maitland, Florida, USA) contains a semirigid integral damper in the longitudinal titanium-alloy rod to reduce stiffness and limits amounts of axial and angular motions at the transition segments [14]. This kind of longitudinal damp-bumping joint dynamically allows ranges-of-motion (ROM) of ±0.4 mm in linear flexion-extension and ±2.25^o^ angular deviation in lateral bending.

From the biomechanical viewpoints, PDS shows a stabilizing effect mainly in flexion/extension and lateral bending but does little to restrict axial rotation on the treated segment [15]. In terms of hybrid device designs, the dynamic part of Dynesys in DTO has a flexible spring cord pretended with adequate spacer length tightening the screw spacer junction to preserve range of motion (ROM) and share mechanical load, thereby shielding loading stress to some extents. In contrast, the damp-bumping part of Isobar affords a rod to decrease stiffness or increase maximal allowable displacement (MAD) for semirigid stabilization. Presently, three inherent problems are concerned in various designs of fixation, which include how a hybrid fixator alters the junctional problem, whether the degenerative grade of transition disc facet affects the selection of a hybrid fixator, and the interfacial stress of screw-vertebra induces device fatigue over long-term use. Therefore, the present study investigates the biomechanical effects of DTO or Isobar device design on the transition and adjacent segments under disc facet degenerative conditions. The longitudinal components of cord/rod stiffness or MAD were varied to investigate the trade-off region (defined as a motion-preserving balance between the transition and adjacent segments) of junctional problem [16]. Owing to the pitfall of device design measurement existing on finite element (FE) analysis, intersegmental motion analysis was supplemented experimentally in the load-transferring survey. The findings of this study will provide insights into the device-related factors of dynamic hybrid fixations associated with surgical complications and suggest an innovative method for surveying pedicle travel mechanism on the PDS under various degenerative conditions.

## 2. Material and Methods

### 2.1. Finite Element Modeling of a Degenerative Lumbosacral Spine

**Figure 1:**
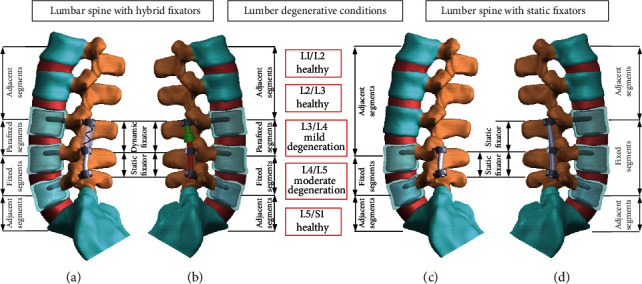
Under a “displacement-controlled” mode, the performances of (a, b) 2 hybrid and (c, d) 2 static lumbar fixation models were normalized by the corresponding degenerative conditions without implantation for comparison.

A lumbosacral model had been established and validated by comparison with the cadaveric and numerical data of intact and degenerative models in our previous study [16]. An intervertebral disc consisting of an annular fibrosus as Mooney-Rivlin hyperelastic composite and nucleus pulposus as a cavity filled with an incompressible fluid was modeled. It was sandwiched between two 1 mm endplates of the neighboring vertebral bodies in this finite element model [17]. The paired articulating surfaces of a healthy facet joint were cautiously prepared to ensure a consistent gap of 0.5 mm in an unloaded neutral position. Nevertheless, the interfacial surface-to-surface contact allowed separation and slippage without friction and only transmitted normal forces during motion [18]. Currently, this study simulated the L4/L5 moderately degenerative disc facet joint as the disc height reduced by 33%, annulus area expanded by 40%, nucleus modulus increased by 66%, and facet gap decreased by 0.3 mm. The L3/L4 disc facet joint was mildly degenerated as the disc height reduced by 15%, annulus area expanded by 16%, nucleus modulus increased by 26%, and facet gap decreased by 0.4 mm (Figure 1) [19]. All main ligaments, including the anterior longitudinal ligament, posterior longitudinal ligament, supraspinous ligament, interspinous ligament, intertransverse ligament, ligamentum flavum, and facet capsular ligament, were modeled as tension only with strain-dependent springs to join their attachments on the adjacent vertebrae. Except for the abdominal muscles, the local muscles were identical to those in the study by Shirazi-Adl et al. [20], which reveals three-dimensional networks of the 5 local muscular groups: quadrates lumborum, iliocostalis, longissimus, iliopsoas, and multifidus distributing onto the vertebral surface with insertion sites cited from the literature [21]. The concentrated loads resulted from the body weight and the abdominal muscles contraction by assigning a compressive force of 500 N, and the flexion, extension, bending, and rotation moments of 10 Nm each upon the lumbosacral top with the L4-L5 static pedicle screws being fixed [16, 22]. A Cartesian coordinate system (*x-y-z*) was established with the origin at the centroid of L5 constrained bottom to describe the 6 degrees of freedom (*df*) motion of the lumbosacral column. A total of 45 ligaments and 46 muscles were simulated symmetrically with respect to *y-z* sagittal plane in this FE analysis [16].

### 2.2. Validation of Isobar Compression-Distraction Stiffness in Mechanical Test for Consecutive FE Modeling

**Figure 2:**
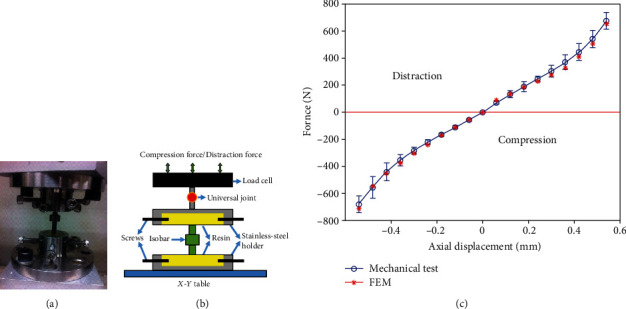
(a) A uniaxial material testing system equipped with a custom-made holder was performed under pure compression and distraction of 700 N load displacement. The distance from Isobar device to epoxy resin surface was fixed with four screws on both sides and mounted by universal joint on a *X*-*Y* table for calculating stiffness under +700 N loading. (b) Numerical Isobar model was set and validated to reveal (c) reaction force averaged 27% lower in compression and 3% higher in distraction than those of experimental results.

To estimate the stiffness of the Isobar damper, a uniaxial material testing system (AG-I, Shimadzu, Japan) equipped with a custom-made holder was adapted under a pure compression and distraction loading (Figure 2(a)). The maximum 700 N axial load at a rate of 0.5 mm/min was set in five repetitive tests to determine the device stiffness from the load displacement curve bilinearly separated into two major slopes (displacement from 0 to 0.4 mm and after 0.4 mm) and transformed into Young's moduli according to the formula: stiffness = (elastic modulus × damper cross − sectional area)/damper length. In compression, the stiffness values averaged 1027 N/mm (ranged, 900-1250 N/mm) during the 0-0.4 mm displacement, and 1724 N/mm (ranged 1050-3000 N/mm) after the 0.4 mm displacement. In distraction, the stiffness values averaged 990 N/mm (ranged 750-1124 N/mm) during the 0-0.4 mm displacement, and 1806 N/mm (ranged 1028-2680 N/mm) after the 0.4 mm displacement. In FE validation, the results adopted in 10 steps with each 0.06 mm displacement revealed the reaction force averaged 27% lower in compression and 3% higher in distraction than those of experimental results (Figures 2(b) and 2(c)). Mesh convergence was conducted with the criteria of displacement < 5% of the standard deviation. The mesh size was set at 2 mm in length for the consecutive FE study.

### 2.3. FE Modeling of DTO and Isobar Devices Fixation

Referring to implantation guide with the same screw diameters for all fixations, the Dynesys cord with 300 N pretension was applied through the cannulated spacer, and the Isobar integral damper was executed with a preset 15° lordotic rod of 5.0 mm in diameter at L3-L4 transition segment after static fixation at L4-L5. The Dynesys spacer simulated as a straight tube (20 mm long, 11.0 mm outer diameter, and 2.0 mm thickness) assumed to be in intimate contact at the screw spacer interfaces. The spring cord stiffness (origin: 650 N/mm) was modified to investigate the trade-off region of junctional problem [16, 22]. The Isobar damp-bumping joint allowed for the same angular rotation with adjustable axial motion of MAD and rod stiffness for comparison [14]. All fixator materials were assumed to have linearly elastic and isotropic material properties throughout, and the von Mises stresses of fixators were compared with the yield strength of corresponding material to assure this assumption. The mesh refinement was controlled locally at the highly stress-concentrated sites and articulating surfaces. Using the aspect ratio and Jacobian check, the quality of all elements was monitored to avoid sharp discontinuities and unrealistically high stress concentrations until excellent monotonic convergence with less than 3% difference of total strain energy was achieved. The nonlinear algorithm with large-deformation formula and direct-sparse solver was utilized by the software Simulation Ed. 2011 (SolidWorks Corporation, Concord, MA, USA). On average, the element numbers of the intact degenerative, static, and hybrid models were about 10,300, 13,000, and 20,000, respectively.

### 2.4. Indices of Comparison

The aforementioned loads and iterative adjustments were applied to control the same value of total disc ROM (by a follower preload) in the models of intact degeneration, 2 static, and 2 hybrid fixations [16, 23–25]. Under a “displacement-controlled” mode, the performance of different lumbar fixations was normalized by the corresponding values of L3/L4/L5 degenerative condition without implantation for comparison (Figure 1). Four indices were chosen to evaluate the behavior of hybrid fixators in terms of disc ROM (Euler angle changes during a given mode of loading), disc stress (average values of the annulus and nucleus stresses), facet contact force (FCF), and stress distribution along screw-vertebral interfaces especially at the transitional level. Furthermore, load-transfer between the cord/rod and vertebrae was investigated experimentally using interpedicular travel (IPT) and interpedicular displacement (ID) measurements [26]. The contact shear stress along the axial line of the pedicle screw denoted the potential for screw loosening in motions. The kinematics of three-dimensional IPT vector and ID acting in tandem revealed the sensitivity of device motion in relation to disc degenerations.

### 2.5. Intersegment Motion Survey of DTO or Isobar in Various Disc Degenerations

**Figure 3:**
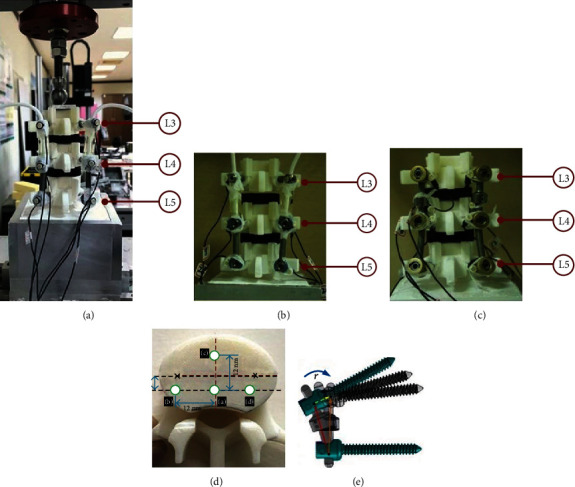
(a) Intersegmental motion behavior was analyzed experimentally on the synthetic model modified from ISO 12189 standards under a “load-controlled” mode. Infrared light emitting diodes were attached to each vertebral body with two landmarkers installed for measuring the 3-dimensional coordinates in (b) DTO and (c) Isobar devices. Initial 2 mm precompression on the assembled construct guaranteed stability and (d) increased axial vertical load of 500 N for overall construct; then, 10 Nm bending moment of flexion or lateral bending was applied to measure (e) interpedicular travel vector (*r*).

In order to evaluate the interpedicular motion of dynamic component in a hybrid fixator, we established a synthetic model using customized discs for anterior-support of vertebra testing blocks modified from the ISO 12189 standards (Figure 3(a)) [27, 28]. The rubber-made intervertebral discs were adapted by 600 or1100 N/mm stiffness (to simulate different grades of disc degeneration) at the transition segment but using unique stiffness of 1600 N/mm at the static segment for a 2-level hybrid device test. Initial 2 mm precompression on the assembled construct guaranteed stability, and axial vertical load increased to 500 N of overall construct for mechanical testing [27]. The position of each vertebra was monitored by infrared light emitting diodes attached to vertebral body with two markers installed for measuring the three-dimensional coordinates (Instron E3000, USA) (Figures 3(b) and 3(c)). The kinematic performance of DTO or Isobar devices at transition segment was evaluated using a motion capture system (Phoenix PTI, Vancouver, BC, Canada). A load-controlled protocol was used to apply 10 Nm bending moment of flexion or lateral bending repeatedly for five cycles (Figure 3(d)) and obtained data on average to measure IPT and ID. The three-dimensional IPT vector *r* ($r=\sqrt{r_{x}^{2}+r_{y}^{2}+r_{z}^{2}}$) in the corresponding reference axes (*x*, *y*, *z*) was calculated to measure the interpedicular motion (Figure 3(e)). The ID was expressed as longitudinal displacement of a flexible cord/rod to indicate the axial stiffness of the device with constraint. All motion behaviors were revealed by calculating the scale difference of IPT or ID magnitude in relation to various disc degenerations. Statistical significance was considered if a *P* value was <0.05 using a paired *t*-test.

## 3. Results

### 3.1. Junctional Problems of Posterior Lumbar Fixators with Original Designs

**Figure 4:**
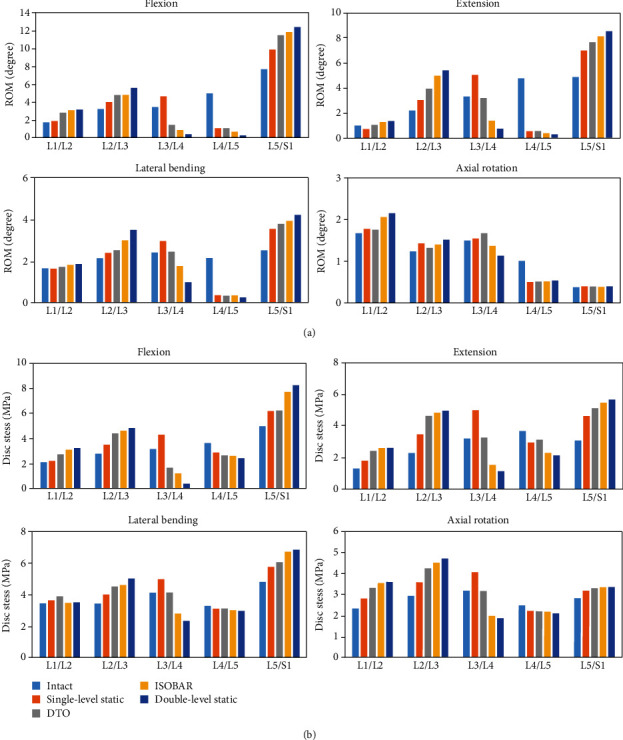
All the DTO, Isobar, 1-level, and 2-level static fixator models revealed various extents of junctional problems at the cephalic segment (L2-L3) more than the caudal one (L5-S1). Both the DTO and Isobar dynamic hybrid fixators had better performance than the 2-level static fixator to increase (a) motion and decrease (b) disc stress at the L3-L4 transition segment for balancing junctional problems.

**Figure 5:**
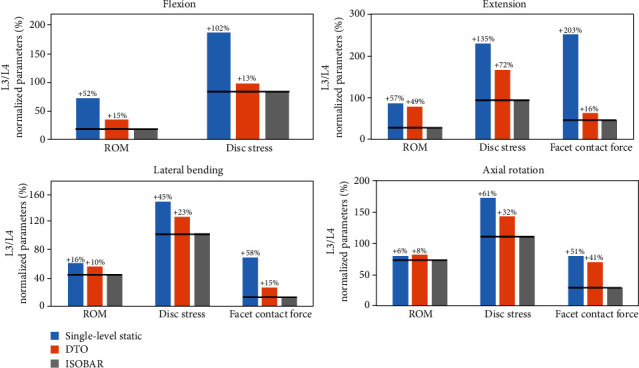
At L3-L4 transition segment, the ROMs, disc stresses, and FCF in the model implanted with dynamic Isobar damper or Dynesys were compared with those of no fixator. The dynamic Isobar damper afforded the decreased FCF, ranging from 15% (bending) to 41% (rotation), more than the Dynesys did.

All the DTO, Isobar, 1-level, and 2-level static fixator models revealed various extents of junctional problems which occurred at the cephalic segment (L2-L3) more than at the caudal one (L5-S1) (Figures 4(a) and 4(b)). Both the DTO and Isobar dynamic hybrid fixators had better performance than the 2-level static fixator in balancing junctional problems. In terms of a normalized 100% degenerative model without instrumentation, the ROMs at L3-L4 transition segment in the DTO group were 15% higher in flexion, 49% higher in extension, 10% higher in lateral bending, and 8% higher in axial rotation, respectively, than those implanted with Isobar integral damper (Figure 5). In the disc stresses at L3-L4 transition segment, the DTO group revealed 13% higher in flexion, 72% higher in extension, 23% higher in lateral bending, and 32% higher in axial rotation, respectively, than the Isobar group. By contrast, the ROMs and disc stresses at L2-L3 supra-adjacent segment of the Isobar model ranged from 2% (rotation) to 24% (extension) higher and from 2% (lateral bending) to 9% (flexion) higher, respectively, than those of the DTO model (Figure 4).

Compared with a degenerative model without instrumentation at the L3-L4 transition segment, the decreased FCF in the model implanted with Isobar integral damper ranged from 6% (rotation) to 57%(extension), from 45% (bending) to 135% (extension), and from 51% (rotation) to 203% (extension), respectively (Figure 5). The decreased FCF in the model implanted with dynamic Dynesys ranged from -2% (rotation) to 37% (flexion), from 22% (bending) to 89% (flexion), and from 10% (rotation) to 187% (extension), respectively, as compared with those with no fixator. In general, the Isobar integral damper constrained ROM and shielded disc stress more than the Dynesys cord did, while the dynamic Isobar damper afforded the decreased FCF, ranging from 15% (bending) to 41% (rotation), more than the Dynesys did at the transition segment.

### 3.2. Biomechanical Performance of DTO within the Trade-Off Region

**Figure 6:**
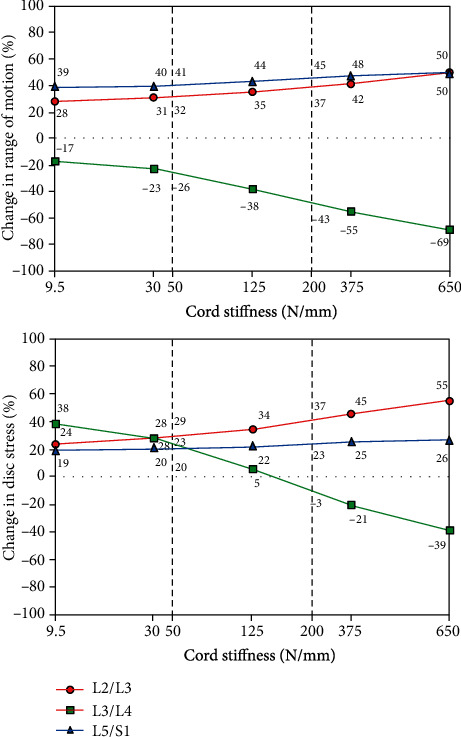
The trade-off region of Dynesys cord stiffness was set as 50-200 N/mm because the convergent value of disc stress decussated around 50 N/mm and approached zero around 200 N/mm. In terms of a 100% degenerative model without instrumentation, the changes of ROM at L3-L4 transition segment ranged from 26% to 43%, and disc stresses ranged from 23% to -3% in flexion to reduce stiffness-related problem.

In the current L3/L4 mild degenerative condition, the trade-off region of Dynesys cord stiffness was set as 50-200 N/mm because the convergent value of disc stress decussated around 50 N/mm and approached zero around 200 N/mm (Figure 6). In terms of a normalized 100% degenerative model without instrumentation, the changes of ROMs ranged from 26% to 43% and disc stresses ranged from 23% to -3% in flexion within the trade-off region at L3-L4 transition segment, as compared with the ROM of 69% and disc stress of 39% at the original 650 N/mm cord stiffness. At the L2-L3 supra-adjacent segment, the changes of ROMs ranged from 32% to 37% and disc stresses ranged from 29% to 37% in flexion, as compared with the ROM of 50% and disc stress of 55% at the original 650 N/mm cord stiffness. Nevertheless, within the trade-off region of cord stiffness, the difference of DTO performance in extension and lateral bending was within 5% and equal in axial rotation at the L3-L4 transition segment. Therefore, under a unique spacer length, the trade-off cord stiffness could induce higher ROMs and lower disc stresses mostly in flexion at the transition segment to reduce the junctional problem, while the trade-off effect was not prominent in extension, lateral bending and rotation.

### 3.3. Biomechanical Performance of Isobar Trade-Off Design

The design effect of MAD versus axial stiffness impact on Isobar performance was compared (Table 1). When the MAD of damper increased from 0.4 mm (*D*1) to 1.2 mm (*D*3), the constraint (changes in percentage) of ROMs at L3/L4 disc were 11% (from -62% to -51%), 8% (-69% to -61%), 9% (-26% to -17%), and 1% (-8% to -7%), and the constraint of L3/L4 disc stresses were 6% (from -55% to -49%), 4% (-65% to -61%), 5% (-32% to -27%), and 4% (-37% to -33%), in flexion, extension, lateral bending, and axial rotation, respectively. When the axial stiffness of damper decreased from 1200 N/mm (*S*1) to 400 N/mm (*S*3), the constraint (changes in percentage) of ROMs at L3/L4 disc were 3% (from -62% to -59%), 3% (-72% to -69%), 8% (-26% to -18%), and 2% (-8% to -6%), in flexion, extension, lateral bending, and axial rotation, respectively, and the constraint of L3/L4 disc stresses displayed little changes (<2%). On average, the MAD induced ROM and disc stress changes (ranged from 9% to 12% in extension and bending) at L2-L3 cephalic adjacent segment more than those at L5-S1 caudal segment. In the current degenerative condition, the MAD effect impact on Isobar performance was more prominent than the axial stiffness effect.

### 3.4. Stress Distribution of Screw-Bone Interfaces in the Dynamic Stabilizers

**Figure 7:**
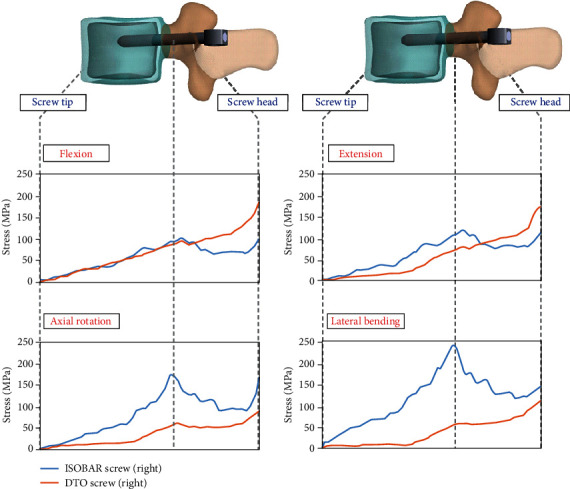
The upper screw-bone interface stress distribution at transition level was shown from the bound screw tip to posterior screw hub of the original dynamic devices. Dynesys screw exhibited a linear pattern of pedicle travel path with peak stress located at the posterior pedicle orifice of screw hub revealing 98% in flexion and 47% in extension, respectively, higher than those of the Isobar integral damper.

The screw-bone interface stress distribution at transition level L3 was shown from the bound screw tip to posterior screw hub under the dynamic fixation of original Isobar or DTO device (Figure 7). The peak stress of Isobar screw was located first at the pedicle eye and second at the junction of pedicle orifice and posterior element under four different motions. These dual peak screw stress in the Isobar damper-bumping device revealed the maximal values of 130% in lateral bending and 100% in axial rotation, which were higher than those of flexion-extension attributing to a 4.5° angular rotation design effect. In contrast, the screw-bone interface stress distribution of Dynesys screw at the transition level exhibited a linear pattern of pedicle travel path with peak stress located at the posterior pedicle orifice of screw hub under 300 N spring cord pretension. The maximal values were 98% in flexion and 47% in extension higher than those of the Isobar integral damper. The stress distribution of Dynesys screws mentioned may predispose to vertical displacement in a relatively weak vertebral bone or device fatigue at the posterior pedicle orifice under a high yielding stress of anterior column in extreme flexion. In contrast, the peak stress of Isobar screw was found at the pedicle eye and decreased within the pedicle in axial rotation and lateral bending beyond the yielding stress of pullout due to the damping mechanism of the shock-absorbing design with stress shielding on the facet joint.

### 3.5. DTO or Isobar Dynamic Motion Behavior in accordance with Various Disc Degenerations

The performance of dynamic motion devices at transition segment was evaluated by calculating the average of IPT and ID in scale (Table 2). When the DTO was installed, the IPT were scaled by 1.18 and 1.29, respectively, in vertical axial load combined with flexion, while the IPT were scaled by 0.90 on the stretching side and by 0.95 on the contraction side in axial load combined with lateral bending. All the IPT induced by DTO revealed no significant differences (*P* > 0.05). On the contrary, the sensitivity of Isobar dynamic performance along the pedicle screw travel path was in close accordance with various disc degenerations. The Isobar revealed significant IPT which were scaled by 0.87 in axial load combined with flexion, and by 0.80 (stretching side) and by 0.50 (contraction side), respectively, in axial load combined with lateral bending (*P* < 0.05). Concerning the ID behavior, the Isobar revealed a significant difference in vertical axial load combined with flexion and contraction side in axial load combined with lateral bending (*P* < 0.05) that corresponded to the design effects of damp axial displacement and ±2.25° angular deviation. The ID behavior of DTO revealed significant borderline differences only in pure axial load or combined with lateral bending that corresponded to the design effects of longitudinal cord/spacer without prominent angular deviation.

## 4. Discussion

The flexibility of dynamic Dynesys cord spacer or Isobar rod-to-rod damper joint was designed in various extents to promote motion preservation and load-sharing effects on the transition segment. We decreased the stiffness of Dynesys cord or Isobar integral damper within “trade-off region” and found the DTO hybrid fixation afforded ROM and disc stress compensation in flexion more physiologically than the Isobar damper rod. The Dynesys trade-off cord stiffness (range, 50-200 N/mm) increased motion at the transition L3/L4 segment to balance the stresses at the adjacent L2/L3 and L5/S1 discs with convergent values of 17% (from 43% to 26%) for ROM and of 26% (from -3% to 23%) for disc stress, respectively, in flexion (Figure 6). Besides, the MAD effect impact on Isobar performance was more prominent than the axial stiffness effect in the current degenerative condition (Table 1). Our previous study showed the stiffer cord was more suitable for the transition disc with more degeneration [16]. Therefore, whether and where to use a dynamic hybrid fixator with the optimal cord/rod designs depended on the bridged disc/facet joint with various degrees of degenerations [9].

In the literature review of FE analysis concerning Dynesys performance, the flexible implant forces depended upon the spring cord stiffness and spacer length (2 mm higher after distraction) to reduce disc stress and posterior annulus bulging [6–9]. The optimal cord stiffness of 50 N/mm fell within the trade-off region of mild degeneration [16]. Currently, the Dynesys trade-off cord spacer improved dynamic motions at the L3-4 transition segment with ROMs increased from 2.7% (rotation) to 12.7% (flexion), disc stresses decreased from 4.1% (flexion) to 12.9% (extension), and FCFs decreased from 4.9% (rotation) to 15.6% (extension) (Figure 6). The spacer length played a critical role in extension and lateral bending to balance the adjacent and transition segments, but had little effect in rotation. Liu et al. investigated the alteration of Dynesys cord pretension with flexion stiffness changing from 19.0 to 64.5 Nm/deg to find FCF increased 35% in extension and higher-stressed pedicle screw in flexion and lateral bending [29]. A lower trade-off cord pretension could afford higher mobility to share load in order to decrease pedicle screw stress in flexion and alleviate FCF in extension. On the contrary, the original 300 N pretended cord made screw spacer linkage contact in extreme flexion, while the high-stressed screw-bone interface might induce device vertical displacement or pullout in weak bones, and further disc/facet degeneration after long-term usage [30, 31].

**Figure 8:**
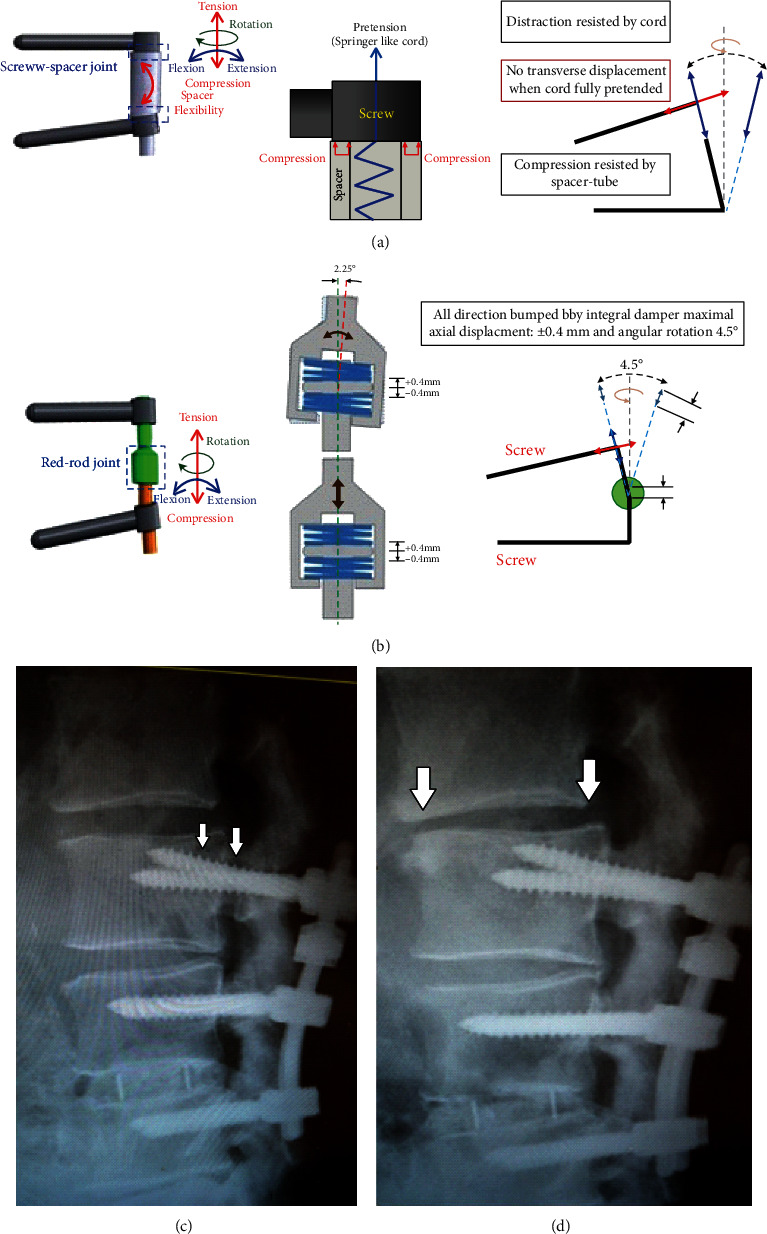
Schematic diagrams illustrated the performances of dynamic hybrid fixators. (a) Dynesys cord pretension made spacer-screw contact but induced high stress at pedicle screw-bone junction. (b) Isobar integral damper provided axial rotation and distraction coupling to afford stress-shielding and reduce loading on the facet joint and pedicle screw. (c) A 70-year-old male patient received Isobar hybrid fixation at L3-4-5 and got back pain at 8 months postoperatively due to screw loosening at L3 (arrow). (d) Subsequently, radicular pain was noticed at four years due to adjacent segment disease at L2-3 (arrow).

The screw spacer linkage acts as a fulcrum leverage with vertebral loads and cord pretension being applied at the two sides. Chien et al. [22] revealed the Dynesys screw spacer contact force was 33% far less than that of the vertebral loads, where there was a sum of 67% cord pretension, muscular contractions, and body weight after extending the cord of 300 N. In this FE degenerative model, a similar cord exhibited a linear IPT pattern with the peak stress imposed at posterior screw hub and induced values of 98% higher in flexion and 47% higher in extension than those of Isobar damper (Figure 7). Intuitively, in clinical viewpoint but beyond FE analysis, the Dynesys cord design in flexion should ensure screw spacer contact (not diverse) under various disc/facet degenerations and lordosis for effective load-shielding to avoid screw loosening [32–34]. Their cord spacer linkage might be incapable of resisting excessive shear loads in potentially unstable spondylolisthesis with a high contact stress imposed on the screw/spacer junction and subsequently induced material fatigue under a heavy vertebral load and extreme flexion [35] (Figure 8(a)).

The Isobar damper joint on a preset lordotic rod allows for 6 *df*-motions to preserve mobility for load sharing at the treated segment. An assembled dampener contained stacks of wear-resistant discs as shock absorbers to reduce axial stiffness (1/3.6 of static rod) [14]. Theoretically, the more mobile damper bumped, the higher ROM and lower disc stress occurred at the transition segment compared to the original set. The current FE study demonstrated the design effect of MAD was more than that of axial stiffness without mentioning angular deviation (Table 1), and the peak stress of Isobar screw at pedicle eye gradually decreased to screw hub induced by the damping mechanism (Figure 7). Theoretically, this damper joint design provided axial rotation and distraction coupling (not permitted by a single-plane hinged screw [36]) to reduce posterior annular bulging indirectly with the location of rotation center shifting anteriorly to be more physiological [14]. The abovementioned motion behavior evident as the sensitivity in accordance with various disc degenerations was demonstrated experimentally to reduce uneven stress exposed at the transitional disc. Besides, the damper design afforded stress shielding to reduce loading on the facet joint and pedicle screw-bone junction during flexion-extension (but less during bending) (Figures 5 and 7). However, the small amount (0.4 mm) of MAD in the Isobar dampener might be adjusted longer in an attempt to avoid pedicle screw departure from intervertebral motion under excessive flexion and improve motion preservation control (Figure 8(b)). In our clinical experience, a patient who underwent an Isobar fixation had initial screw loosening and developed symptomatic adjacent segment disease later (Figures 8(c) and 8(d)).

The IPT and ID metrics denoted the relative motion of adjacent pedicle screws at the transition segment. The optoelectric tracking of three-dimensional kinematic behavior has been conducted to find that a longer spacer in Dynesys increased ROM and decreased FCF [9]. We adapted this kind of direct tracking to demonstrate the IPT path related to the extent of disc degeneration in Isobar damper joint more than in the Dynesys cord spacer (Table 2). IPT is not measured in the vicinity of rotation center but is related to a summation of translational and rotational motion of spinal unit based on the pedicle screw tracking. Additional measurements of the longitudinal cord/rod dynamic components can be obtained not only through kinematic tracking of vertebral bone alone (as in the FE analysis) but also experimentally assessing the sensitivity of device motion behaviors under various disc degenerations. Therefore, an intuitive translation between the biomechanical test and FE analysis would allow for more sensitive detections of various PDS performance in clinical relevance.

Discrepancies between numeric and experimental results exist owing to the simplification of spinal fixator design in the FE model or difficulty in maintaining longitudinal rod or cord spacer exactly at the same lordotic angle during consecutive experimental tests. Initial preload guaranteed the stability on an instrumented assembly to reduce half the von Mises stress values [27]. The current interpedicular motion survey adapted a capture system on synthetic models to measure the scale difference of IPT or ID in relation to disc degenerations. The load shared by posterior hybrid implants varies depending on its anterior disc stiffness characteristics under the applied motion; thus, good agreements between the FE prediction and experimental measurement can assure a dynamic loading performance. A comparable bending movement of the upper body, such as heavy lifting with forward flexion, led to a high risk of mechanical fatigue [28]. The internal loads (i.e. axial load, bending, and torsional moments) on the cord/rod might be compared to make the simulation models more reliable [37].

### 4.1. Limitations of the Current Study

With respect to the model limitations, our study assumed the changes in disc-facet strength concurrently without involving lordotic curve progression, facet hypertrophy, endplate sclerosis, osteophytes, and annular tears. Regarding the designs of spinal implant, hyperelastic behavior of cord spacer was not considered, and with only one length of spacer support for FE analysis. Meanwhile, the screw stress may be underestimated because of the lack of a screw thread and simplification of screw configuration. The loosening of bone screw and screw spacer interfaces were focused as major failure modes in dynamic stabilization systems, but the complex interfacial slippage was not easily modeled for the sake of efficiency and convergence of highly nonlinear simulation [38]. This FE analysis would not mention about several situations of destabilized spine fixed with hybrid implants, fatigue failure of Isobar screw rod, and loading condition was only applied to the displacement-controlled method [39]. If the load-controlled method is applied to evaluate nonfusion spinal implants, the variation of ROM effects for the stabilized segment is remarkable [23]. Owing to the FE analytic controversy with the above assumptions, intersegmental motion survey with a load-controlled method was supplementary to quantify the biomechanical alteration with different disc stiffness at the transition level particularly in flexion and bending motions for a Dynesys or Isobar system. There were still some limitations for the present synthetic testing model, such as lacks of posterior elements, facet joint, bone screw interfacial stress evaluation, and without considering ligament constraint, lordotic angle changes, and pelvic parameters. However, the kinematic sensitivity related to disc degeneration for both dynamic stabilizers was found through this standardized in vitro test. Further clinical and experimental studies should be conducted to confirm the findings of this study and long-term use of hybrid fixation.

## 5. Conclusion

Dynamic hybrid fixators can protect transition segment but may induce kinematic and mechanical compensation to adjacent segments which was a trade-off problem of the fixed segment. For the Dynesys component, the highly stressed interface of bone screw and screw spacer makes them potentially pullout and fatigue failure. The Isobar damper joint should adjust the maximal allowable displacement with less stress occurring at screw vertebral junction for dynamic fixator use.

## Figures and Tables

**Table 1:** Design effects of axial stiffness (S) versus maximal allowable displacement (MAD) on the Isobar performance (in terms of 100% intact degenerative model without instrumentation).

Performance	Design parameter		Flexion (%)			Extension (%)			Lateral bending (%)			Axial rotation (%)		
			L2/3	L3/4	L5/S1	L2/3	L3L4	L5/S1	L2/3	L3/4	L5/S1	L2L3	L3L4	L5/S1
ROM	Axial stiffness (kN/mm)	*S*1 = 1.2	57	-62	54	104	-72	41	40	-26	76	15	-8	-14
		*S*2 = 0.8	↓			↓			↓			↓		
		*S*3 = 0.4	57	-59	53	101	-69	39	31	-18	76	15	-6	-12
	MAD (mm)	*D*3 = 1.2	53	-51	52	92	-61	38	31	-17	75	15	-7	-11
		*D*2 = 0.8	↑			↑			↑			↑		
		*D*1 = 0.4	57	-62	54	104	-69	41	40	-26	76	15	-8	-14
Disc stress	Axial stiffness (kN/mm)	*S*1 = 1.2	51	-55	22	96	-65	22	34	-32	40	52	-37	13
		*S*2 = 0.8	↓			↓			↓			↓		
		*S*3 = 0.4	49	-55	22	95	-65	22	22	-29	39	52	-35	13
	MAD (mm)	*D*3 = 1.2	47	-49	52	84	-61	38	22	-27	35	48	-33	10
		*D*2 = 0.8	↑			↑			↑			↑		
		*D*1 = 0.4	51	-55	54	96	-65	41	34	-32	40	52	-37	13

**Table 2:** The interpedicular travel (IPT) and interpedicular displacement (ID) of DTO and Isobar at transition segment were evaluated by calculating scale difference.

Load condition	Device	IPT				ID			
		*A* (mm)	*B* (mm)	*B*/*A*	*P* value	*C* (mm)	*D* (mm)	*D*/*C*	*P* value
Axial load	DTO	0.17 ± 0.10	0.20 ± 0.07	1.18	0.28	−0.01 ± 0.03	−0.07 ± 0.05	7	<0.001
	Isobar	0.95 ± 0.07	0.58 ± 0.23	0.61	<0.001	0.10 ± 0.07	0.02 ± 0.05	0.20	0.02
Axial+flexion	DTO	0.14 ± 0.05	0.18 ± 0.10	1.29	0.18	−0.01 ± 0.05	−0.04 ± 0.02	4	0.13
	Isobar	0.84 ± 0.07	0.75 ± 0.06	0.87	0.01	0.45 ± 0.11	0.11 ± 0.06	0.24	<0.001
Axial+bending (stretching side)	DTO	0.52 ± 0.10	0.47 ± 0.04	0.90	0.21	0.07 ± 0.02	0.11 ± 0.01	1.57	0.01
	Isobar	1.00 ± 0.06	0.80 ± 0.06	0.80	0.01	0.25 ± 0.06	0.25 ± 0.03	1.00	0.43
Axial+bending (contraction side)	DTO	0.66 ± 0.12	0.63 ± 0.09	0.95	0.36	−0.38 ± 0.04	−0.44 ± 0.01	1.16	0.02
	Isobar	1.02 ± 0.08	0.51 ± 0.09	0.50	<0.001	−0.22 ± 0.06	−0.06 ± 0.02	0.27	<0.001

Note: the stiffness of 600 N/mm (*A* and *C*) and 1100 N/mm (*B* and *D*), respectively, were used to simulate different grades of mild degeneration at L3/4. The stiffness of 1600 N/mm was used to stimulate moderate degeneration at L4/5 for all models in the mean time.

## Data Availability

The finite element and in vitro test data used to support the findings of this study are included within the article.
